# Insidious APOL1 Kidney Disease: A Manifestation of APOL1-Associated Pregnancy Complications on Nephron Endowment?

**DOI:** 10.3390/cells14171373

**Published:** 2025-09-03

**Authors:** Timur Azhibekov, Leslie A. Bruggeman

**Affiliations:** 1 Division of Neonatology, Cleveland Clinic Children’s, Cleveland, OH 44195, USA; azhibet2@ccf.org; 2 Department of Inflammation & Immunity, Cleveland Clinic Research, Cleveland, OH 44195, USA; 3 School of Medicine, Case Western Reserve University, Cleveland, OH 44106, USA

**Keywords:** APOL1, chronic kidney disease, genetics, kidney development, low birth weight, preeclampsia, prematurity

## Abstract

The mechanism of kidney injury associated with apolipoprotein L1 (*APOL1*) risk variants has remained elusive. Complicating this issue is the broad clinical spectrum of APOL1 kidney disease, which has engendered speculation that this reflects multiple mechanisms of kidney injury. APOL1 kidney disease can be rapid in onset with heavy proteinuria, associated with viral infections and categorized pathologically as collapsing focal segmental glomerulosclerosis. Alternatively, APOL1 kidney disease also may present as an insidious, slowly progressive disease, with less proteinuria but losses in glomerular filtration rate and with varied pathology. In addition to APOL1 kidney disease, *APOL1* risk variants are also associated with preeclampsia and other conditions related to placental insufficiency. The outcome of these APOL1-associated pregnancy complications frequently results in prematurity and low birth weight, both of which are known risk factors for hypertension and kidney disease later in life due to reduced nephron endowment. The significance of *APOL1* risk variants on pregnancy complications that predispose to kidney disease should not be overlooked as a central mechanism of APOL1 kidney disease, especially the insidious forms, which are difficult to distinguish from the spectrum of kidney disease attributable to prematurity and low birth weight. If low nephron endowment is a causal mechanism behind some forms of APOL1 kidney disease, this may have an impact on clinical trials evaluating drugs directly inhibiting *APOL1*, since in these instances, ongoing *APOL1* expression may not be driving podocyte loss and progressive kidney dysfunction.

## 1. Introduction

A breakthrough in 2010 identified the genetic basis for the enhanced risk for chronic kidney disease (CKD) associated with African ancestry [1,2,3]. Polymorphisms in the gene for apolipoprotein L1 (APOL1, gene name: *APOL1*) constituting two allelic variants known as G1 and G2 were associated with CKD when inherited as a recessive trait. These risk variants were enriched in individuals of recent sub-Saharan West African ancestry due to their beneficial protection against lethal parasitic infections but are absent in other demographic groups. Over the past 15 years, how *APOL1* risk variants cause injury to the kidney and how best to treat individuals with APOL1 kidney disease have been areas of intense research efforts. An early experimental attempt to develop a small animal model for human *APOL1* expression serendipitously discovered that *APOL1* risk alleles are also expressed in the placenta and cause preeclampsia [4], another disease with excess risk in mothers of African descent [5,6,7]. Several human cohort studies have now confirmed *APOL1* high-risk genotypes in the infant predispose the mother to preeclampsia, resulting in infant prematurity and low birth weight [8,9,10,11,12,13]. The potential for this pregnancy complication to have a significant impact on the presentation of APOL1 kidney disease and a comparison to the current disease paradigm of direct effects of APOL1 on kidney cells are the focus of this review.

## 2. APOL1 and CKD Risk

The current theory is APOL1 kidney disease pathogenesis is a two-hit mechanism [14]. The first hit, the *APOL1* high-risk genotype, consists of any combination of two copies of the G1 or G2 variant alleles. The high-risk genotype, however, is only a genetic susceptibility and alone is insufficient to cause CKD. Individuals with high-risk genotypes remain disease-free until a second hit stressor is encountered. The CKDs most strongly associated with *APOL1* risk variants are triggered by viral infections, such as human immunodeficiency virus-associated nephropathy (HIVAN) [15,16,17,18,19], coronavirus disease-associated nephropathy (COVAN) [20,21,22,23,24], and others [25]. Mechanistically, immune responses to the viral infection, which involve secretion of high levels of interferons and other pro-inflammatory cytokines, induce *APOL1* expression in podocytes [26]. This high level of *APOL1* expression subsequently causes podocyte loss by either cell death or detachment leading to the prototypical pathology seen in APOL1 kidney disease: focal segmental glomerulosclerosis (FSGS). Exactly how APOL1 biochemically engages cellular pathways to cause podocyte loss is not fully understood, and numerous causal pathways have been implicated [27,28,29], in addition to potential complicating issues relating to splice variants, cell types, and haplotype effects [30,31,32]. This lack of a unified understanding of the function of APOL1 in kidney cells, in combination with a limited understanding of the potential range of disease-causing second hits, has fueled speculation that the varied presentation of APOL1 kidney diseases is attributable to a complex interaction of genetic, cellular, and environmental factors [30].

There are, however, some common points of understanding in the penetrance and phenotype variability in the presentation of APOL1 kidney disease [30,33]. First, the two-hit model described above is based on APOL1 kidney diseases in which the second hit stressor is known, those caused by viral infections. These APOL1 kidney diseases are overt and relatively rapid in onset, with nephrotic range proteinuria and with the severe FSGS pathology of glomerular collapse (Table 1). These infection-associated presentations have the highest odds ratios for CKD risk in cohort studies (reviewed in [33]). In cases of HIV-associated nephropathy, suppressing the viral infection with antiretroviral therapy, thereby eliminating the second hit from the pathogenic equation, can restore kidney function and pathology [34]. Details on the mechanism of the viral second hit were further revealed in experiments [35,36,37] and human observational reports [38]. The common theme is that the anti-viral immune response, not the virus itself, is likely the critical factor for disease induction since administration of interferons alone can similarly cause kidney disease. However, it also has been shown in model systems using interferon stimulation to induce *APOL1* expression that kidney injury can be mitigated using agents to directly suppress APOL1 [37,39]. Therefore, kidney injury appears to be caused by a combination of *APOL1* risk-variant expression in the setting of a strong interferon response. It is important to note pre-clinical experiments supporting the development of therapeutics to treat APOL1 kidney disease, including those currently in clinical trials, were based on this two-hit, interferon-dependent model [37,39,40,41].

The second common point of understanding in the phenotype variability is that most presentations of APOL1 kidney disease do not have an obvious second hit [2,42,43,44,45,46,47,48]. These CKDs are insidious, with unclear disease onset, characterized functionally by a subclinical decline in estimated glomerular filtration rate (eGFR) but without severe proteinuria and with a more varied pathological picture (Table 1). The insidious presentations have lower odds ratios in cohort studies compared to the viral-induced overt presentations. This failure to understand the more common, insidious presentation of APOL1 kidney disease has resulted in speculation on various potential mechanisms. One mechanism previously proposed by several groups expands on the two-hit interferon model [49,50]. In this potential scenario, instead of a single strong and sustained interferon-producing event, there are multiple, but less severe and transient events, such as infection with common cold or flu viruses, resulting in short-term and limited episodes of podocyte losses. Thus, in individuals with *APOL1* high-risk genotypes, the life-long accumulation of typical anti-viral responses could amass excessive podocyte losses with age. This would then result in a more gradual functional decline but still with progression to a clinical CKD diagnosis faster or more frequently than individuals with low-risk genotypes. There is no direct evidence for this proposed mechanism, but future studies using available mouse models may be helpful to test this hypothesis. In addition, all forms of CKD can be impacted by common co-morbidities, such as hypertension, diabetes or metabolic abnormalities, and environmental and lifestyle factors, such as smoking and alcohol use, which may synergize to hasten functional declines by mechanisms independent of *APOL1* expression. However, an alternative potential explanation of the insidious presentation may lie in the association of *APOL1* risk alleles with pregnancy complications.

**Table 1:** Comparison of the CKD presentations for APOL1 kidney disease with CKDs associated with prematurity and low birth weight.

	Overt Presentation	Insidious Presentation	CKD from Prematurity or Low Birth Weight
CKD onset	severe/rapid	subclinical/chronic	subclinical/chronic
Clinical presentation	nephrotic range proteinuria	eGFR decline	eGFR decline
	eGFR decline	hypertension	hypertension
		proteinuria	microalbuminuria
Pathology	FSGS/collapsing FSGS	Nephrosclerosis, microcysts	Nephrosclerosis,
	Podocytopathy [18,20,51]	FSGS, IFTA [44,52,53]	FSGS, IFTA [54,55]
Diagnoses	HIVAN [15,16,17,18]	HTN-attributed CKD or ESKD [2,42,43,44,45]	HTN-attributed CKD [56]
	COVAN [20,21,22,23,24]	non-diabetic ESKD [46,47,48]	glomerular disease/nephritis interstitial nephritis [57,58]
	Interferon therapy use [38]	primary CKD progression (LN, MN, SCN) [59,60,61,62,63,64]	primary CKD progression (IgAN, PKD, MN, MCD, DN) [65,66,67,68]
	FSGS [2,16,43,69]		secondary to AKI [70,71]
CKD odds ratio	17–89 (African ancestry) [33]	2–11 (African ancestry) [33]	1.2–6 (all races) [57,72,73,74,75,76]

AKI, acute kidney injury; CKD, chronic kidney disease; COVAN, coronavirus disease-associated nephropathy; DN, diabetic nephropathy; ESKD, end stage kidney disease; FSGS, focal segmental glomerulosclerosis; HIVAN, human immunodeficiency virus associated nephropathy; HTN, hypertension; IFTA, interstitial fibrosis tubular atrophy; IgAN, IgA nephropathy; LN, lupus nephritis; MCD, minimal change disease; MN, membranous nephropathy; PKD, polycystic kidney disease; SCN, sickle cell nephropathy.

## 3. APOL1 and Risk for Preeclampsia, Prematurity, and Low Birth Weight

As noted above, animal models of human *APOL1* expression revealed the potential contribution of *APOL1* risk variants to preeclampsia [4,77]. These studies compared mice transgenic for the non-disease *APOL1* G0 with the disease-associated G1 or G2 alleles, including one mouse model that replicated the endogenous expression of *APOL1* [77]. Since mice do not have a gene equivalent to human *APOL1*, the transgenic mice expressing either the G1 or G2 allele are a genocopy for humans homozygous for either G1 or G2, and thus these studies essentially examined the effects of *APOL1* high-risk genotypes. Both studies documented typical features of preeclampsia in the mice expressing either the G1 or G2 alleles but not in the G0 transgenic mice, including elevated blood pressure and profound outcomes on the morbidity and mortality of both the pregnant female (eclamptic seizures) and in the offspring pups, including perinatal mortality and low birth weight. An interesting caveat of these observations was that the risk of preeclampsia was dependent on the *APOL1* genotype of the fetus not the mother, implicating an important contribution of the paternal *APOL1* genotype to preeclampsia risk. *APOL1* is abundantly expressed in the placenta [78], with high levels of expression in trophoblasts [4], key cell types required for the development of the placental vasculature and maintenance of the maternal–fetal blood barrier [79]. Preeclampsia is caused by the failure of spiral artery remodeling to low resistance/high flow blood vessels resulting in under perfusion of the placenta. This leads to placental insufficiency, a common pathogenic event in various disorders that can result in fetal growth restriction and low birth weight, still birth, and prematurity. The function of APOL1 in the placenta and the mechanism by which *APOL1* risk variants lead to placental insufficiency and pregnancy complications are unknown. In speculation, a possible mechanism for APOL1-associated preeclampsia may be similar to the *APOL1* risk variant cytotoxicity described for podocyte cell death in CKD [14]. Trophoblast migration into the myometrium and invasion of maternal spiral arteries is required to initiate their vascular remodeling to adequately supply blood flow to the placenta and support the growing fetus [80]. If *APOL1* risk variant expression similarly results in trophoblast cell death, there may be too few trophoblasts to induce spiral artery remodeling, setting in motion the well-known mechanism for preeclampsia and placental insufficiency [80].

Similar to CKD, the incidence of prematurity and low birth weight in African Americans is significantly greater than that of European Americans (Table 2). Current United States national vital statistic birth data report African Americans have a greater incidence of prematurity (<37 weeks), low birth weight (<2500 g), and preeclampsia [81]. Similar numbers also apply to other populations of Black African ancestry, with reports of elevated risk of preeclampsia or low birth weight in Caribbean Hispanics and in south and west African countries [82,83,84,85]. Cohort studies of African Americans, other African diasporas, and sub-Saharan African communities found fetal *APOL1* high-risk genotype predisposes the mother to risk for preeclampsia and infants born with low birth weight [8,9,10,11,12,13]. The risk for preeclampsia, prematurity, or low birth weight was specific to the fetal *APOL1* genotype and did not associate with the maternal *APOL1* genotype [10,11,12,86]. When excluding other psychosocial and economic confounders that can contribute to pregnancy complications, the fetal *APOL1* high-risk genotype still accounts for a significant portion of the excess risk of preeclampsia in African ancestry compared to European ancestry [10]. Although preeclamptic pregnancies frequently result in preterm birth, in a large cohort of term preeclamptic pregnancies, infants with the high-risk *APOL1* genotype showed altered fetal growth velocity and had significantly higher risk of being small for gestational age (<10th percentile in birth weight) [9]. This suggests that in less severe cases of preeclampsia and independent of prematurity, these pregnancies are characterized by an adverse intrauterine environment secondary to placental insufficiency. These observations together implicate the fetal *APOL1* risk genotype as a significant risk factor for both prematurity and low birth weight.

## 4. Pregnancy Complications, Nephron Endowment, and CKD Risk

Prematurity and low birth weight are well-established risk factors for CKD, decreased eGFR, and hypertension in both children and adults [57,72,74,76,91,92]. The CKDs associated with prematurity and low birth weight have similar features to the insidious presentation of APOL1 kidney disease (Table 1). These CKDs can have varied diagnoses but frequently there is no identifiable cause [56]. They can become evident in childhood or adulthood, typically with unclear disease onset, but they are progressive with a common disease pathology of glomerulonephritis or FSGS [57,58]. Odds ratios for CKD risk range similarly to the insidious presentations of APOL1 kidney disease. Connections between *APOL1* genotype, kidney disease, and prematurity or low birth weight have been examined by multiple groups using the NEPTUNE, CKiD, and CureGN consortia [93,94,95]. Prematurity was significantly more common in children and adults with *APOL1* high-risk genotypes, typically with a glomerular disease diagnosis, lower eGFR at study entry, and faster eGFR decline compared to an *APOL1* low-risk genotype.

The mechanism for CKD caused by prematurity and low birth weight originates in kidney development (reviewed in [96]). Nephron endowment is determined at birth and establishes the maximum functional capacity of the kidneys. Since a majority of nephrogenesis takes place in the third trimester, prematurity can significantly interrupt kidney development and reduce total nephron numbers. Low birth weight and smallforgestationalage status are determined by both the length of gestation and the quality of the intrauterine environment. Therefore, in addition to prematurity, an adverse intrauterine environment can both reduce birth weight and kidney size or maturation [97].

Brenner originally proposed that low nephron endowment can be the origin of kidney disease and hypertension in later life [98]. This work arose from observations on the classic experimental model known as five-sixths nephrectomy, in which after partial nephrectomy, the remnant kidney is subject to maladaptive responses [99]. To compensate for low nephron numbers, the existing nephrons hyperfilter and hypertrophy, reducing sodium excretion and altering glomerular hemodynamics. These adaptations lessen the ability of the kidney to withstand stress (i.e., functional kidney reserve), the outcome of which is hypertension and risk for CKD. Experimental rodent models controlling nephron number or podocyte loss demonstrated podocyte attrition was a central component to nephron functional decline [54,100,101]. Since glomerular podocytes are terminally differentiated and do not undergo mitosis, the increase in glomerular size requires existing podocytes to stretch over the hypertrophied glomerulus causing mechanical strain on the podocyte. This strain places the podocyte at risk for detachment or cell death, further reducing podocyte density in the glomerulus, which perpetuates continued podocyte loss and causes the typical scar formation of FSGS.

Biopsy and autopsy studies have linked prematurity and low birth weight with low glomeruli numbers, increased glomerular volume, and low podocyte density per glomerulus in subjects with and without a diagnosed CKD [58,102,103]. Recent studies have begun examining *APOL1* genotype effects on nephron numbers and podocyte densities. In mouse models of human *APOL1* expression (including mouse models replicating native *APOL1* expression), *APOL1* risk alleles were associated with larger glomerular volumes (indicating hypertrophy) and lower podocyte densities (indicating podocyte loss) compared to mice expressing *APOL1* G0 [4,104]. These were the same transgenic mouse models that also exhibited preeclampsia and low birth weights with *APOL1* risk allele expression. In human studies, examination of kidneys from healthy transplant donors observed *APOL1* high-risk genotype donors had larger glomeruli and fewer podocyte numbers compared to low-risk genotypes [105]. In a larger cohort of African Americans without a clinical CKD diagnosis, *APOL1* high-risk genotypes similarly were associated with losses of podocytes and increases in glomerular volumes with age [106]. Neither of the human studies directly linked *APOL1* genotype and low nephron endowment with a birth complication, specifically prematurity and low birth weight, and future studies making this connection with birth history are needed. Continued experiments in the existing *APOL1* transgenic mice should be useful in testing this mechanism, and newer imaging and morphometric methods have made estimating nephron numbers in humans more feasible [107].

## 5. Summary

**Figure 1:**
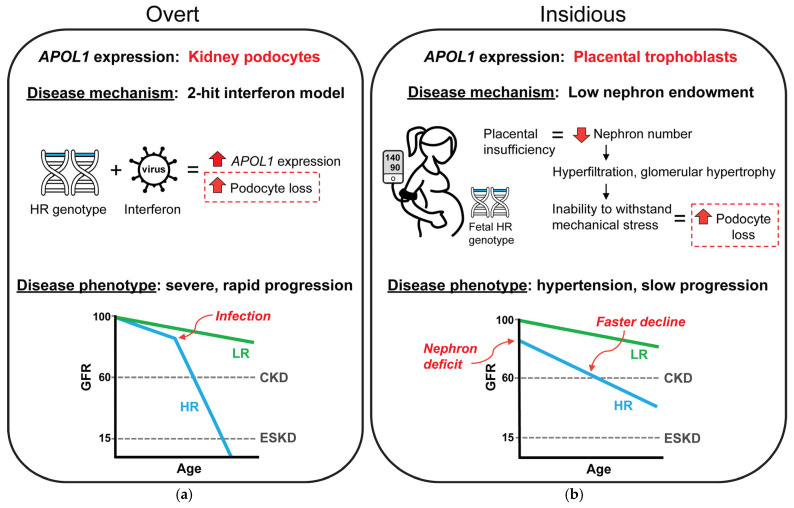
Proposed mechanistic difference between the varied presentations of APOL1 kidney disease. (**a**) The best understood presentation of APOL1 kidney disease is an overt, rapidly progressive CKD that is typically associated with a viral infection or other chronic inflammatory state. These inflammatory conditions are associated with high interferon levels which induce *APOL1* expression in kidney podocytes resulting in dramatic podocyte losses and nephrotic range proteinuria. The overt presentation best fits the proposed two-hit model for APOL1 kidney disease pathogenesis. (**b**) Less understood is the insidious presentation of APOL1 kidney disease, a subclinical disease that is characterized by slowly progressive losses in GFR, frequently with hypertension. This presentation of APOL1 kidney disease is similar to CKD that can develop as a consequence of prematurity and low birth weight through a mechanism caused by low nephron endowment. A developmental deficit in nephron numbers predisposes kidneys to gradual, chronic podocyte loss from compensatory mechanisms related to countering the mechanical strain during filtration. Thus, with the evidence for *APOL1* expression in placental trophoblasts and the association of fetal *APOL1* high-risk genotypes with preeclampsia and low birth weight, it is possible that a similar CKD mechanism for podocyte loss may underlie the insidious presentation of APOL1 kidney disease. In this potential mechanism, podocyte losses would not require ongoing expression of *APOL1* in the kidney but are founded in the effect of *APOL1* expression during pregnancy and also would not require a second hit to cause CKD. HR, high-risk *APOL1* genotype; LR, low-risk *APOL1* genotype, CKD, chronic kidney disease, ESKD, end stage kidney disease, GFR, glomerular filtration rate.

In the varied presentation of APOL1 kidney disease, the two-hit model of pathogenesis best fits the overt CKDs associated with viral infections, whereas the insidious presentation may be explained, in part, by low nephron endowment caused by pregnancy complications (Figure 1). In both potential mechanisms the endpoint of the pathogenic process culminates in loss of podocytes, resulting in similar FSGS pathology. In the overt presentation, podocyte loss is driven by ongoing *APOL1* expression in the kidney; however in the insidious presentation, podocyte loss is driven by mechanical strain in the glomerulus. In the insidious presentation, the APOL1-dependent event that caused CKD was expression of *APOL1* in the placenta, and thus this mechanism would not require any direct effect of *APOL1* expression after pregnancy. It is possible to still consider the low nephron endowment mechanism a two-hit model. Low nephron endowment at birth is frequently considered a susceptibility event since the reduced nephron number limits the functional reserve of the kidney to withstand disease stressors or even normal aging. Thus, low nephron endowment can be a first-hit susceptibility to other more common insults associated with CKD risk, such as hypertension and diabetes. The magnitude, frequency, or compounding of these second-hit CKD risk factors may further accelerate nephron loss and result in variabilities in CKD presentation, severity, or progression.

If low nephron endowment is a contributor to some presentations of insidious APOL1 kidney disease, the current anti-APOL1 drugs in clinical testing may be ineffective in these cases since ongoing *APOL1* expression is not driving podocyte loss and the ensuing proteinuria and eGFR decline. These new anti-APOL1 therapeutics represent a long-needed advance for kidney diseases that have previously lacked any specific therapeutics and disproportionally impact underserved populations [108]. However, if low nephron endowment does contribute to insidious APOL1 kidney disease, then these clinical trials may have some non-responders. Based on the existing studies examining *APOL1* risk genotypes and prematurity and low birth weight (summarized in Table 2), approximately 20% of individuals with *APOL1* high-risk genotype experienced significant birth complications that could be the underlying cause for their CKD. Establishing a direct cause–effect relationship connecting (1) *APOL1* risk genotype with pregnancy complications, (2) pregnancy complications with low nephron endowment, and (3) low nephron endowment with CKD may be challenging and may remain only an association. However, prematurity and low birth weight are well-recognized as significant contributors to CKD risk later in life, and evidence is growing associating *APOL1* genotypes with pregnancy complications that can cause low nephron endowment. For future cohort studies and clinical trials, it will be difficult to separate CKDs caused by APOL1-associated birth complications from those caused by ongoing *APOL1* expression in the kidney unless information is collected on birth history.

## 6. Conclusions

The high incidence and significance of pregnancy-related birth complications caused by *APOL1* risk genotypes are potentially an under-recognized contributor for CKD risk. The varied presentations of APOL1 kidney disease may in part result from placental *APOL1* expression and its effect on nephron endowment, since the insidious presentation of APOL1 kidney disease has many similarities to kidney disease attributed to prematurity and low birth weight. If the mechanism of low nephron endowment underlies insidious APOL1 kidney disease, clinical trials testing anti-APOL1 drugs may not be efficacious in these cases since ongoing *APOL1* expression in the kidney would not be driving podocyte injury and loss. As recently discussed, clinical assessment of birth history is underutilized in evaluating CKD risk [109]. If *APOL1* genotyping is used to guide management of future CKD risk, an accompanying evaluation of birth history could provide a more comprehensive understanding of the long-term impact of *APOL1* genotype and treatment options.

## Figures and Tables

**Table 2:** Impact of *APOL1* high-risk genotype on incidence and risk of preeclampsia and low birth weight in the United States.

	Preeclampsia		Low Birth Weight or Small for Gestational Age		
	Incidence (% Births)	Odds Ratio	Incidence (% Births)	Odds Ratio	Citation
European Americans	7.1%	(reference)	9.4%	(reference)	[81]
African Americans	9.9%	1.3–1.6 *	12.9–14.8%	1.7–2.6 *	[81,87,88,89,90]
African Americans with *APOL1* high-risk genotype	15–22%	1.7–3.6 **	19.3–20.2%	2.4–5.5 **	[8,9,10,11]

* Comparison to European Americans. ** Comparison to African Americans with *APOL1* low-risk genotypes.

## Data Availability

No new data were created for this manuscript.

## Abbreviations

The following abbreviations are used in this manuscript:

AKI	acute kidney injury
APOL1	Apolipoprotein L1
COVAN	coronavirus disease-associated nephropathy
CKD	chronic kidney disease
CKiD	Chronic Kidney Disease in Children Study
CureGN	Cure Glomerulonephropathy Consortium
DN	diabetic nephropathy
eGFR	estimated glomerular filtration rate
ESKD	end stage kidney disease
FSGS	focal segmental glomerulosclerosis
GFR	glomerular filtration rate
HIVAN	human immunodeficiency virus-associated nephropathy
HTN	hypertension
IFTA	interstitial fibrosis tubular atrophy
IgAN	IgA nephropathy
LN	lupus nephritis
MCD	minimal change disease
MN	membranous nephropathy
NEPTUNE	Nephrotic Syndrome Study Network
PKD	polycystic kidney disease
SCN	sickle cell nephropathy
